# PFOA accumulation in the leaves of basil (*Ocimum basilicum* L.) and its effects on plant growth, oxidative status, and photosynthetic performance

**DOI:** 10.1186/s12870-024-05269-0

**Published:** 2024-06-14

**Authors:** Fabrizio Pietrini, Anna Wyrwicka-Drewniak, Laura Passatore, Isabel Nogués, Massimo Zacchini, Enrica Donati

**Affiliations:** 1 Research Institute on Terrestrial Ecosystems (IRET), National Research Council of Italy (CNR), Via Salaria km 29.300, Monterotondo Scalo, Roma, 00015 Italy; 2 Faculty of Biology and Environmental Protection, Department of Plant Physiology and Biochemistry, University of Lodz, ul. Banacha 12/16, Lodz, 90-237 Poland; 3 Institute for Biological Systems (ISB), National Research Council of Italy (CNR), Via Salaria km 29.300, Monterotondo Scalo, Roma, 00015 Italy

**Keywords:** Antioxidant activity, Chlorophyll fluorescence, Perfluoroalkyl substances (PFASs), Photochemical reflectance

## Abstract

**Background:**

Perfluoroalkyl substances (PFASs) are emerging contaminants of increasing concern due to their presence in the environment, with potential impacts on ecosystems and human health. These substances are considered “forever chemicals” due to their recalcitrance to degradation, and their accumulation in living organisms can lead to varying levels of toxicity based on the compound and species analysed. Furthermore, concerns have been raised about the possible transfer of PFASs to humans through the consumption of edible parts of food plants. In this regard, to evaluate the potential toxic effects and the accumulation of perfluorooctanoic acid (PFOA) in edible plants, a pot experiment in greenhouse using three-week-old basil (*Ocimum basilicum* L.) plants was performed adding PFOA to growth substrate to reach 0.1, 1, and 10 mg Kg^− 1^ dw.

**Results:**

After three weeks of cultivation, plants grown in PFOA-added substrate accumulated PFOA at different levels, but did not display significant differences from the control group in terms of biomass production, lipid peroxidation levels (TBARS), content of α-tocopherol and activity of ascorbate peroxidase (APX), catalase (CAT) and guaiacol peroxidase (POX) in the leaves. A reduction of total phenolic content (TPC) was instead observed in relation to the increase of PFOA content in the substrate. Furthermore, chlorophyll content and photochemical reflectance index (PRI) did not change in plants exposed to PFAS in comparison to control ones. Chlorophyll fluorescence analysis revealed an initial, rapid photoprotective mechanism triggered by PFOA exposure, with no impact on other parameters (F_v_/F_m_, ΦPSII and qP). Higher activity of glutathione S-transferase (GST) in plants treated with 1 and 10 mg Kg^− 1^ PFOA dw (30 and 50% to control, respectively) paralleled the accumulation of PFOA in the leaves of plants exposed to different PFOA concentration in the substrate (51.8 and 413.9 ng g^− 1^ dw, respectively).

**Conclusion:**

Despite of the absorption and accumulation of discrete amount of PFOA in the basil plants, the analysed parameters at biometric, physiological and biochemical level in the leaves did not reveal any damage effect, possibly due to the activation of a detoxification pathway likely involving GST.

**Supplementary Information:**

The online version contains supplementary material available at 10.1186/s12870-024-05269-0.

## Background

The presence of chemical compounds derived from human activities in environmental matrices is a growing concern due to their toxic effects on living organisms and the ecosystem. In this context, the growth of wild and cultivated plant species is increasingly affected by the occurrence of xenobiotics in the environment [1]. In particular, crop plants grown in soil contaminated by toxic compounds or irrigated with contaminated water can accumulate and transfer harmful substances along the food chain [2]. This process, known as bioaccumulation, represents a significant risk to human and animal health.

Particular concern is raised by compounds recently introduced by the chemical industry, as they can interact with biochemical processes and pose a hazard to human and ecosystem health. The chemicals, whose toxicity levels are currently under examination, have been identified as Compounds of Emerging Concern (CEC) [3, 4]. In recent years, there has been significant interest in endocrine-disrupting chemicals, pharmaceuticals, personal care products, microplastics and flame retardants, among others [5, 6]. Many of these substances are still lacking any regulatory standards.

Flame retardants are a group of chemicals that are increasingly used to protect against accidental fires by reducing the flammability of materials such as plastics and synthetic polymers [7]. The most representative flame retardants are halogen and phosphorus-based compounds, with brominated and fluorinated compounds being suspected of toxic effects on humans and the environment [8, 9].

Perfluoroalkyl substances (PFASs) have been employed for decades as firefighting foams and in various other industrial applications, including aviation lubricants, paints, pharmaceuticals, and cosmetics [10], thanks to their chemical properties such as hydrophobicity and oleophobicity. Furthermore, this category of chemicals exhibits significant chemical stability, as evidenced by their resistance to hydrolysis, photolysis, biodegradation, and metabolism [11]. The extensive utilisation of these compounds, particularly in the recent past, coupled with their physico-chemical properties, has led to the widespread pollution of various environments, including the extreme Arctic ice [12]. Background concentrations of PFOA in soils range from 0.01 to 123.6 µg kg^− 1^, while can reach even 50,000 µg kg ^− 1^ in contaminated soils [13]. Perfluoroalkyl substances are presently identified in numerous plant and animal species, both in terrestrial and aquatic ecosystems, ultimately affecting humans through the food chain [14–16]. Perfluorooctane sulfonate (PFOS) and perfluorooctanoic acid (PFOA) are two compounds comprising eight carbon atoms which are typically found among perfluoroalkyl substances (PFASs) in soil (µg/kg dw) and water sources, including surface, marine, and drinking water (ng/L) [17, 18].

It has been demonstrated that PFASs can be taken up by plants from soil and surface water, and accumulated in roots and above-ground organs. This accumulation occurs through both the symplastic and apoplastic routes, as reported by Felizeter et al. [19], Wen et al. [20], and Wang et al. [21]. The toxicity of PFAS in plants is well documented, suggesting that the onset of oxidative stress is the primary biological process induced by the presence of PFOS and PFOA. This is characterised by an imbalance between the production and disposal of reactive oxygen species (ROS) [22–24]. The oxidative burst can result in the alteration of expression of numerous genes involved in physiological processes like photosynthesis, hormone signalling, and energy metabolism. This can result in a reduction in growth, which is typically the most visible symptom [22, 25–27]. Therefore, the characterisation of both the oxidative stress status and the antioxidant response is considered a key aspect for evaluating the impact of PFOA on plants [28]. The accumulation of PFAS compounds in agricultural crops is a matter of serious concern due to their possible bioaccumulation and transfer along the food chain involving livestock and humans. In addition to direct toxicity in plants grown in PFAS-contaminated substrates, there is evidence of hazardous levels of PFASs, particularly PFOS and PFOA, being detected in the edible parts of crops [29]. Various factors, at chemical, agronomical, and environmental level, have been reported to affect the absorption and partitioning of PFASs in the organs of cultivated plants. Among these factors, the shorter length of the carbon chain of PFAS molecules was found to be associated with a higher absorption and translocation ability in *Arabidopsis thaliana* plants compared to longer chained compounds, which mainly remained adsorbed to the roots [30]. At the agronomical level, cultivation methods, plant species, and substrate characteristics were commonly indicated to affect the concentration of PFAS molecules in plant tissues. With regard to plant species, both cereals and vegetables were found to accumulate PFASs in their organs, with PFOA concentrations typically higher than PFOS [31–34]. In cereals, straw is reported as the preferred site of PFAS accumulation, with wheat plants exhibiting a higher capacity to absorb these compounds compared to oat and maize, respectively [31, 32]. Furthermore, both authors indicated a lower PFAS concentration in grains than in vegetative organs of cereal plants cultivated in PFAS-enriched soils. Perfluorooctanoic acid and PFOS were predominantly found in the roots of the plants, with Felizeter et al. [19, 35] reporting similar results in hydroponically grown lettuce, tomato, cabbage, and zucchini plants exposed to 14 different PFASs. Finally, Ghisi et al. [29] reported that environmental factors such as irradiance, temperature, and humidity could potentially affect the absorption and translocation of PFASs in plants by driving stomatal opening and transpiration.

The present study, dealing with an experimental greenhouse trial, was aimed to study the effects of PFOA, administered to plants through growth substrate, on the growth and the photosynthetic performances of a widely cultivated food plant, namely common basil (*Ocimum basilicum* L.). As previously reported, the impairment of the oxidative status is highlighted as one of the most relevant toxic effects exerted by PFAS in plants. Therefore, in this work, the oxidative stress status and the antioxidative response were specifically addressed. In this context, the oxidative stress markers (i.e. thiobarbituric acid reactive substances; TBARS), as well as the non-enzymatic (i.e. total phenolic content; TPC and α-tocopherol content), the enzymatic antioxidant plant response (i.e. ascorbate peroxidase; APX, catalase; CAT, guaiacol peroxidase; POX), and the activity of the detoxifying enzyme glutathione S-transferase (GST) were analysed in basil leaves, in parallel with the analysis of PFOA accumulation. Perfluorooctanoic acid was chosen as one of the two most abundant PFAS in the environmental matrices [18], with concentrations generally higher than those of PFOS in cultivated plants [31–34]. Perfluorooctanoic acid concentrations tested were selected based on similar studies [25, 36, 37], taking into account the concentrations of PFAS compounds in contaminated sites [13]. To the best of our knowledge, no literature examines the effects of the exposure to PFASs of basil, a plant species widely used in fresh recipes and with economic significance in the perfume, pharmaceutical, and medical industries [38].

## Materials and methods

### Plant material and growth conditions

Three-week-old basil (*Ocimum basilicum* L.) plants, supplied from a local nursery, were transplanted in 3.5 L plastic pots, closed at bottom with plastic bag to avoid leaching and filled with a universal plant growth substrate (Select-Klasmann-Deilmann GmbH, Germany, Table S1). After one week of adaptation to greenhouse condition (natural photoperiod, with mean (night-day) temperatures of 17–25 °C and relative humidity of 60–80%), homogeneous plants for size and physiological conditions were selected and randomly chosen to arrange four theses, each consisting of five pots (replicates) with one plant per pot: not spiked substrate (Control, C), substrate + 0.1 mg PFOA Kg^− 1^ dw (PFOA 0.1), substrate + 1 mg PFOA Kg^− 1^ dw (PFOA 1), substrate + 10 mg PFOA Kg^− 1^ dw (PFOA 10). PFOA was added to substrate as aqueous solution (previously dissolved in methanol, final solution containing no more than 1% of methanol) at the beginning of the experiment. No methanol was added to the control soil, as in the preliminary experiments on plantlets, and in a previous study [39], we found no effect of methanol at this concentration on plants. During the cultivation period (three weeks), the water regime was maintained by daily restoring the amount of water lost for evapo-transpiration adding purified tap water directly in the pot. At the end of the treatment period, before sampling, physiological assays focused on chlorophyll fluorescence imaging analysis and on the assessment of the photochemical reflectance index (PRI) and chlorophyll content were performed. Plants were then harvested, carefully washed with water, and separated into organs (roots, stem and leaves) for the fresh weight measurement and the collection of leaf samples for PFOA quantification and biochemical analyses (immediately frozen in liquid nitrogen and stocked at -80 °C). A part of the sample was used for dry weight determination and weighted after 72-hour drying in an oven at 60 °C.

### PFOA detection and quantification in plants

#### Extraction and clean-up

Frozen plant material was ground in a mill under liquid nitrogen, lyophilized in a vacuum freeze dryer (Labconco, Kansas City, MO, USA) and stored at − 20 °C until use. About 0.2 g of each lyophilized sample was weighed, added into a 50 mL polypropylene (PP) centrifugal tube and spiked with 25 ng of ^13^C_4_-PFOA as internal standard. After vortexing and equilibrating for half an hour, 5 mL of 1% (v/v) formic acid in ACN solution were added to each tube. Samples were vortex-mixed again for 1 min, placed in an ultrasonic bath for 10 min and finally centrifuged for 10 min (10,000 rpm, 10 °C). The extraction was repeated twice more and the supernatants were combined in a 15 mL PP tube, in order to reduce the volume extract to 1 mL under a gentle nitrogen stream. Afterwards, the concentrated extract was subjected to clean-up by solid phase extraction. To this end, the extract was first diluted with 9 mL of water and then loaded onto the Oasis HLB cartridge, pre-conditioned with 5 mL of MeOH and 5 mL of water in turn. After the sample loading, the cartridge was sequentially eluted with 4 mL of MeOH and 4 mL of a solution containing 1% (v/v) NH_4_OH in MeOH. After the clean-up process, the eluate was evaporated to dryness under N_2_ stream, reconstituted in 1 mL of MeOH and lastly filtered through a 0.22 μm nylon filter before the analysis.

The above procedure was used for the PFOA determination in plants treated with PFOA solutions at 1 mg L^− 1^ and 10 mg L^− 1^. In order to quantify PFOA in plants treated with PFOA solution at 0.1 mg L^− 1^, 0.2 g of lyophilized sample were spiked with 2.5 ng of internal standard. Subsequently the extraction and the clean-up step (according to the procedure previously described), the resulting extract was evaporated to 0.1 mL under N_2_ stream and filtered through a 0.22 μm nylon filter prior to the injection.

#### UPLC – MS analysis

Chromatographic analysis was carried out by an Acquity UPLC H-Class Bio system from Waters (Millford, MA, USA), equipped with a quaternary pump, a sample manager, an autosampler, a column temperature controller and a PDA. PFOA in the basil extracts was determined using an Acquity UPLC BEH C18 column (50 × 2.1 mm id, 1.7 μm particle size) maintained at 40 °C and equipped with an Acquity UPLC BEH C18 VanGuard pre-column (1.7 μm, 2.1 × 5 mm). Chromatographic elution was performed in gradient mode by using the following solvents system: 2 mM ammonium acetate buffer pH 6 containing 5% (v/v) MeOH (Phase A) and MeOH (Phase B). Gradient elution was composed of the following steps: 0–2 min, 5–60% B; 2–5 min, 60% B; 5–10 min 100% B (hold 2 min). The flow rate was 0.3 mL min^− 1^ and the injection volume was 5 µL.

Quantification of PFOA was achieved coupling on-line the UPLC system to an ion-trap mass spectrometer (LXQ-MS System, Thermo Scientific) via an electrospray ionization (ESI) source operating in negative ion mode. ESI source and ion-trap parameters were optimized to get the highest sensitivity.

Instrumental settings included scan range from 150 to 700 m/z; heater temperature, 290 °C; nitrogen sheath gas flow, 5 arbitrary units; capillary temperature, 240 °C; capillary voltage, -12 V; spray voltage, 3.1 kV. PFOA was detected in full-scan mode using extracted ion chromatograms from the parent ion (m/z = 413). The absence of the target compound in plant tissue was preliminarily checked and procedural blanks were included during the analyses.

#### Method validation

Method validation was performed by determining linearity, sensitivity, precision and accuracy. A matrix-matched calibration curve, containing 1,2,3,4-^13^C_4_-PFOA as internal standard, was constructed in order to minimize the interferences of the matrix constituents on the detector response. Matrix-matched calibration standards in the concentrations ranged between 2.5 and 150 mg/L (*n* = 7) were prepared by diluting the intermediate standard solution of native PFOA with the blank plant extract. Each matrix-matched standard solution was spiked with 25 mg/L of 1,2,3,4-^13^C_4_-PFOA. The curve was linear over the concentration range studied and the correlation coefficient was found to be 0.9998. Instrumental detection limit (IDL) was evaluated on a signal-to-noise ratio of 3 in MeOH and it was found to be 0.8 mg/L. Method detection limit (MDL) and method limit of quantification (MLQ) were assessed as the lowest spiked concentration showing a signal-to-noise ratio of 3 and 10 in the chromatogram, respectively. Notably MDL and MLQ were 0.89 ng/g_dw_ and 2.67 ng/g_dw_, respectively. Precision of the entire method was assessed by determining the relative standard deviations (RSD) of replicate analyses (*N* = 6) of a spiked extract, during the same day for repeatability (RSD ≤ 1) and on three different days for reproducibility (RSD ≤ 2). Accuracy was evaluated by studying the recovery of PFOA from plant samples fortified at different levels (10, 20 and 50 ng/g_dw_) before the extraction. Recovery values were higher than 95% for all samples.

### Pigment content measurement

Measurements of total chlorophyll content were performed by the chlorophyll meter readings (SPAD-502, Minolta Camera Co., Osaka, Japan). The measure was taken from four fully developed leaves per plant. SPAD readings were taken from the widest portion of the leaf lamina, while avoiding major veins. The four SPAD readings were averaged to represent the SPAD value of each plant. SPAD values were converted to chlorophyll content (µg cm^− 2^) using the equation reported in Cerovic et al. [40]: Chlorophyll content = (99 x SPAD)/(144 - SPAD).

### Photochemical reflectance index determination

Leaf reflectance, measured in two narrow wavelength bands centred close to 531 nm and 570 nm, was used for Photochemical Reflectance Index (PRI) determination, which is calculated as PRI = (R_531_ − R_570_)/(R_531_ + R_570_), where R is the reflectance at each wavelength [41]. The measurements were performed on the same leaves used for the SPAD readings with a portable instrument PlantPen PRI 210 (Photon Systems Instruments, Drásov, Czech Republic) that directly recorded the PRI values. The data were extracted and processed with FluorPen Software (Photon Systems Instruments, Drásov, Czech Republic).

### Imaging of chlorophyll fluorescence

The maximal quantum yield of photosystem II (PSII) photochemistry (F_v_/F_m_), the effective quantum yield of PSII photochemistry (ΦPSII), the quantum yield of regulated non-photochemical energy loss (ΦNPQ), the quantum yield of non-regulated non-photochemical energy loss (ΦNO) and the photochemical quenching (qP) were measured on the last fully expanded leaf using a chlorophyll fluorescence imaging (MINI-Imaging-PAM, Walz, Germany). Leaves were dark adapted for at least 30 min before determining F_0_ and F_m_ (minimum and maximum fluorescence, respectively). The F_v_/F_m_ value was calculated as (F_m_- F_0_)/F_m_. Subsequently, leaves were adapted to a photosynthetic photon flux density (PPFD) of 440 µmoles m^− 2^ s^− 1^ for at least 5 min to reach a steady-state condition and then the parameters ΦPSII, ΦNPQ, ΦNO and qP, were measured as reported by Di Baccio et al. [42] and Kramer et al. [43].

### Total phenolic content analysis

The extraction of total phenolic compounds was performed using 2 g of plant material with 80% methanol (5 mL). The amount of extracted total phenolic compounds was determined with the Folin–Ciocalteu reagent as previously described [44]. Each analysis was performed in duplicate for each extract. The gallic acid was used as the standard and the total phenolic compounds were expressed as mg of gallic acid equivalents (GAE) per g of fresh weight.

### Preparation of extracts from leaf tissues

Whole leaves were homogenized (1:5 w/v) in an ice-cold mortar using 50 mM sodium phosphate buffer, pH 7.0, containing 0.5 M NaCl, 1 mM EDTA, 0.5% polyvinylpyrrolidone (PVP) and 1 mM sodium ascorbate. The slurry was filtered through two layers of Miracloth. The filtrate of homogenized leaves was then centrifuged (15,000 *g* × 15 min). After centrifugation, the supernatant was collected and ascorbate peroxidase (APX), catalase (CAT), guaiacol peroxidase (POX), glutathione S-transferase (GST) activities as well as protein content and degree of lipid peroxidation were measured.

### Enzyme assay

APX activity [EC 1.11.1.11] was assayed following the oxidation of ascorbate to dehydroascorbate at 265 nm (ε = 13.7 mM^− 1^ cm^− 1^) according to Nakano and Asada [45] with some modifications. The assay mixture contained 50 mM sodium phosphate buffer pH = 7.0, 0.25 mM sodium ascorbate, 25 µM H_2_O_2_ and the enzyme extract (5–10 µg protein). The addition of H_2_O_2_ started the reaction. The obtained values were compared with those of another reaction mixture without the enzyme extract to correct for non-enzymatic oxidation of ascorbate. The enzyme activity was expressed in µmol ascorbate min^− 1^ mg^− 1^ protein.

CAT activity [EC 1.11.1.6] was measured spectrophotometrically according to Dhindsa et al. [46]. A reaction mixture composed of 50 mM sodium phosphate buffer (pH = 7.0), 15 mM H_2_O_2_ and the enzyme extract (5–10 µg protein) was used. The decomposition of H_2_O_2_ (ε = 45.2 mM^− 1^ cm^− 1^) was measured at 240 nm. CAT activity was expressed in µmol Η_2_Ο_2_ min^− 1^ mg^− 1^ protein.

POX activity [EC 1.11.1.7] was assayed with guaiacol according to Chance and Maehly [47], with modifications. A linear increase in absorbance at 470 nm was observed due to the formation of tetraguaiacol (TG; ε = 26.6 mM^− 1^ cm^− 1^). The reaction mixture contained 49 mM sodium acetate buffer (pH 5.6) 5 mM guaiacol, 15 mM H_2_O_2_, and the enzyme extract (15–25 µg protein). The enzyme activity was expressed in mmol TG min^− 1^ mg^− 1^ protein.

The total GST activity [EC 2.5.1.18] was determined with 1-chloro-2,4-dinitrobenzene (CDNB) according to Habig et al. [48] with some modification. GST catalyses the conjugation of L-glutathione (GSH) to CDNB to form 2.4-dinitrofenylo-S-glutathione which absorbs at 340 nm (2.4-DNFSG; ε = 9.6 mM^− 1^ cm^− 1^). The reaction solution contained 100 mM potassium phosphate buffer pH 6.25, 0.75mM CDNB, 30mM GSH and enzyme extract (50 µg protein). The enzyme activity was expressed in nmol 2.4-DNFSG min^− 1^ mg^− 1^ protein.

All enzyme activity assays were performed spectrophotometrically using a Unicam UV 300 UV-Visible spectrometer (Unicam Limited, Cambridge United Kingdom) at 25 °C.

### Protein content

The total soluble protein content was determined according to Bradford [49] with standard curves prepared using bovine serum albumin by spectrophotometer (Helios Gamma, Thermo Spectronic, Cambridge, UK).

### Degree of lipid peroxidation

The content of lipid peroxides was estimated spectrofluorometrically (F-2500 Fluorescence Spectrophotometer; Hitachi, Limited, Tokyo Japan) according to Yagi [50] with some modifications, by measuring the content of 2-thiobarbituric acid reactive substances (TBARS). The content of lipid peroxides was calculated in terms of 1,1,3,3- tetraethoxypropane (TEP), which was used as a standard, and expressed in nmol TEP g^− 1^ fresh weight.

### Determination of α-tocopherol

Whole leaves were homogenized (1:5 w/v) in an ice-cold mortar using 50 mM sodium phosphate buffer, pH 7.0, containing 0.5 M NaCl, 1 mM EDTA and 1 mM sodium ascorbate. Crude homogenate obtained after filtration was assayed for α-tocopherol content according to modified method of Taylor et al. [51]. After saponification of the sample with KOH in the presence of ascorbic acid, α- tocopherol was extracted with n-hexane. Fluorescence of the organic layer was measured at 280 nm (excitation) and 310 nm (emission) using a F-2500 Fluorescence Spectrophotometer (Hitachi, Limited, Tokyo Japan). The content of α-tocopherol was expressed as µg α-tocopherol g^− 1^ fresh weight of the original plant tissue.

### Statistical analysis

The data reported refer to a representative experiment with five replicates. After the check for normal distribution by using the SPSS (Chicago, IL, USA) software tool, data were processed by one-way ANOVA. Statistical significance of the mean data was assessed by Holm-Sidak test, except for total chlorophyll content and Photochemical Reflectance Index (PRI) in which Tukey test was used. Statistical data treatment was performed by using the SPSS (Chicago, IL, USA) software tool. A summary of ANOVA data regarding biometric and physiological parameters (Table S2) and PFOA content and biochemical parameters (Table S3) is reported in the Supplementary materials.

## Results and discussion

The growth of basil plants exposed to different concentrations of PFOA was unaffected, regardless of the amount of PFOA added to the substrate (Table 1 and Table S4). Furthermore, no statistical difference in biomass production was observed in the three sampled organs as a result of the plant exposure to PFOA. The results of this study are consistent with those of Zhou et al. [25], who found no alteration in wheat plant growth when exposed to similar concentrations of PFOA in soil in pots. However, higher concentrations of PFOA were found to induced a significant reduction in growth. Similarly, Li et al. [22] and Li and Li [23] reported no biomass reduction in lettuce exposed to PFOA under hydroponic conditions. Fan et al. [26] observed a reduction in growth in *Arabidopsis thaliana* seedlings grown in MS medium with 20 µM PFOA, with severe growth inhibition observed at 200 µM PFOA. In contrast, Pietrini et al. [39] observed no growth impairment in the duckweed species *Lemna minor* following treatment under laboratory conditions with low concentrations of PFOA (2, 20 and 200 µg L^− 1^) in the growth solution. Overall, the data reported in the literature highlight the still fragmentary information on PFOA toxicity in plants and demonstrate the variable effects of PFOA in plants, mainly due to the plant species and growth medium used. In this regard, the fate, transport, and transformation of PFAS in soil and water environments have been reviewed [52, 53].

**Table 1:** Biomass (g dw) of different organs of basil plants at the end of three weeks of growth in pots filled with soil with different PFOA concentrations (0 mg Kg^− 1^, Control; 0.1 mg Kg^− 1^, PFOA 0.1; 1 mg Kg^− 1^, PFOA 1; 10 mg Kg^− 1^, PFOA 10). In each column, similar letters represent statistically not different values (mean values ± S.E., *n* = 5)

	Plant biomass (g dw)		
Treatments	Leaves	Stem	Roots
Control	10.91 (± 0.53) ^a^	2.96 (± 0.25) ^a^	2.69 (± 0.32) ^a^
PFOA 0.1	10.99 (± 0.43) ^a^	3.21 (± 0.18) ^a^	2.72 (± 0.18) ^a^
PFOA 1	10.53 (± 0.41) ^a^	3.45 (± 0.14) ^a^	3.33 (± 0.28) ^a^
PFOA 10	11.35 (± 0.29) ^a^	3.57 (± 0.13) ^a^	3.05 (± 0.24) ^a^

Despite the absence of any observed effect of PFOA on plant growth, the accumulation of PFOA in basil leaves was detected in all plants grown on the PFOA-enriched substrate (Table 2). Notably, this accumulation was found to be proportional to the concentration of PFOA initially present in the pots, with its content increasing in the leaves of plants exposed to higher doses of PFOA. According to Lechner and Knapp [54], who argued that the uptake of PFOA by plants is related to the tenside nature of PFOA and therefore to a certain solubility in water, the accumulation of PFOA in basil leaves is likely the result of the uptake of PFOA by the rooting system and its translocation to the leaves by the transpiration stream in the xylem vessels. It should be noted that other potential sources of interference such as atmospheric deposition and leaf contamination by irrigation cannot be claimed because the experiment was conducted in a greenhouse and irrigation with PFOA-free water was applied precisely to the substrate surface. Nevertheless, the amount of PFOA detected in basil leaves is consistent with that commonly reported in the literature, although differences in plant species, PFOA concentration and substrate make comparison among studies difficult. In a similar study, Blaine et al. [33] observed that radish and celery plants accumulated PFOA in their edible parts with concentrations ranging from 60 to 600 ng g^− 1^ dw, respectively, when grown under greenhouse conditions in soils amended with municipal or industrial biosolids. In leaves of cucumber plants grown in greenhouses in soils amended with PFOA concentrations similar to those used in the present study (i.e. 0.2 and 5 mg PFOA Kg ^− 1^ soil dw), Du et al. [37] found PFOA levels of approximately 13 and 300 ng g^− 1^ dw, highlighting the ability of this plant species, among others, to accumulate and translocate PFOA and concluding that the use of this plant species in PFOA-contaminated soils is not recommended. Furthermore, the accumulation of PFOA in plants has also been studied using hydroponics as a growing system, thus avoiding the interaction between soil properties and the chemical compound. Li et al. [22] found PFOA accumulation of 35.1 and 316.7 ng g^− 1^ dw in the leaves of lettuce plants grown in the nutrient solution supplemented with 5 and 50 µg L^− 1^ PFOA, respectively, i.e. concentrations much lower than those used in the present study. Additionally, limited PFOA accumulation by horticultural crops under hydroponic conditions has also been reported in other studies. In this regard, Dal Ferro et al. [55] reported that lettuce and spinach leaves from plants grown in water spiked with 500 ng L^− 1^ PFOA accumulated 3 and 3.8 ng g^− 1^ dw, respectively. Therefore, there is currently no clear evidence that plants are more efficient in translocating absorbed PFOA to aerial parts in hydroponics than in soil cultivation. As highlighted by Felizeter et al. [56], at least for lettuce, only the transfer of PFASs from the substrate to the roots was 1–2 orders of magnitude higher under hydroponic conditions, while the transfer to the foliage was similar in hydroponic and soil-grown plants.

**Table 2:** Perfluorooctanoic acid (PFOA, ng g^− 1^ dw), TBARS (TEP, nmol g^− 1^ fw), α-Tocopherol (α-TOC, µg g^− 1^ fw), and total phenolic concentration (TPC, µg gallic acid g^− 1^ fw) in the leaves of basil plants at the end of three weeks of growth in pots filled with soil with different PFOA concentrations (0 mg Kg^− 1^, Control; 0.1 mg Kg^− 1^, PFOA 0.1; 1 mg Kg^− 1^, PFOA 1; 10 mg Kg^− 1^, PFOA 10). In each column, different letters represent statistically different values (mean values ± S.E., *n* = 5; Holm-Sidak test, *P ≤* 0.05; nd, not detected)

Treatments	PFOA	TBARS	α-TOC	TPC
Control	nd	1.82 (± 0.14) ^a^	9.26 (± 0.88) ^a^	371.5 (± 27.4) ^a^
PFOA 0.1	2.68 (± 0.36) ^c^	1.78 (± 0.24) ^a^	8.58 (± 1.81) ^a^	316.8 (± 30.2) ^ab^
PFOA 1	51.8 (± 4.7) ^b^	1.71 (± 0.24) ^a^	10.38 (± 0.49) ^a^	305.1 (± 12.2) ^ab^
PFOA 10	413.9 (± 41) ^a^	1.43 (± 0.19) ^a^	15.29 (± 4.88) ^a^	257.8 (± 2.45) ^b^

The accumulation of PFAS in edible plant parts represents a significant concern due to the potential risk associated with their ingestion through the human diet. In this regard, the European Food Safety Authority [57] has reported that the Tolerable Weekly Intake (TWI) for PFOA should be 6 ng kg^− 1^ of body weight. In this context, estimating the risk associated with the dietary intake of basil leaves grown in the PFOA-contaminated soil of the present study is not an easy task due to the fragmentary information on the daily consumption of basil leaves. However, assuming the basil daily intake reported by Ciriello et al. [58], and taking into account the PFOA level occurring in the soil [13], it can be stated that, for basil plants grown in the soil with PFOA at background level, the risk for human consumption is negligible (plants grown in 0.1 mg kg^− 1^ in our trial), while, for basil plants grown in moderate to highly contaminated soil, the risk for human consumption can be considered high or very high (plants grown in 1 and 10 mg kg^− 1^ in our trial, respectively). Nevertheless, when dealing with the PFOA toxicity, we have to consider that “For PFOA, the total contribution from the non-food sources, mainly indoor exposure, could be as high as 50% compared to the estimated average dietary exposure to PFOA” [59].

**Fig. 1:**
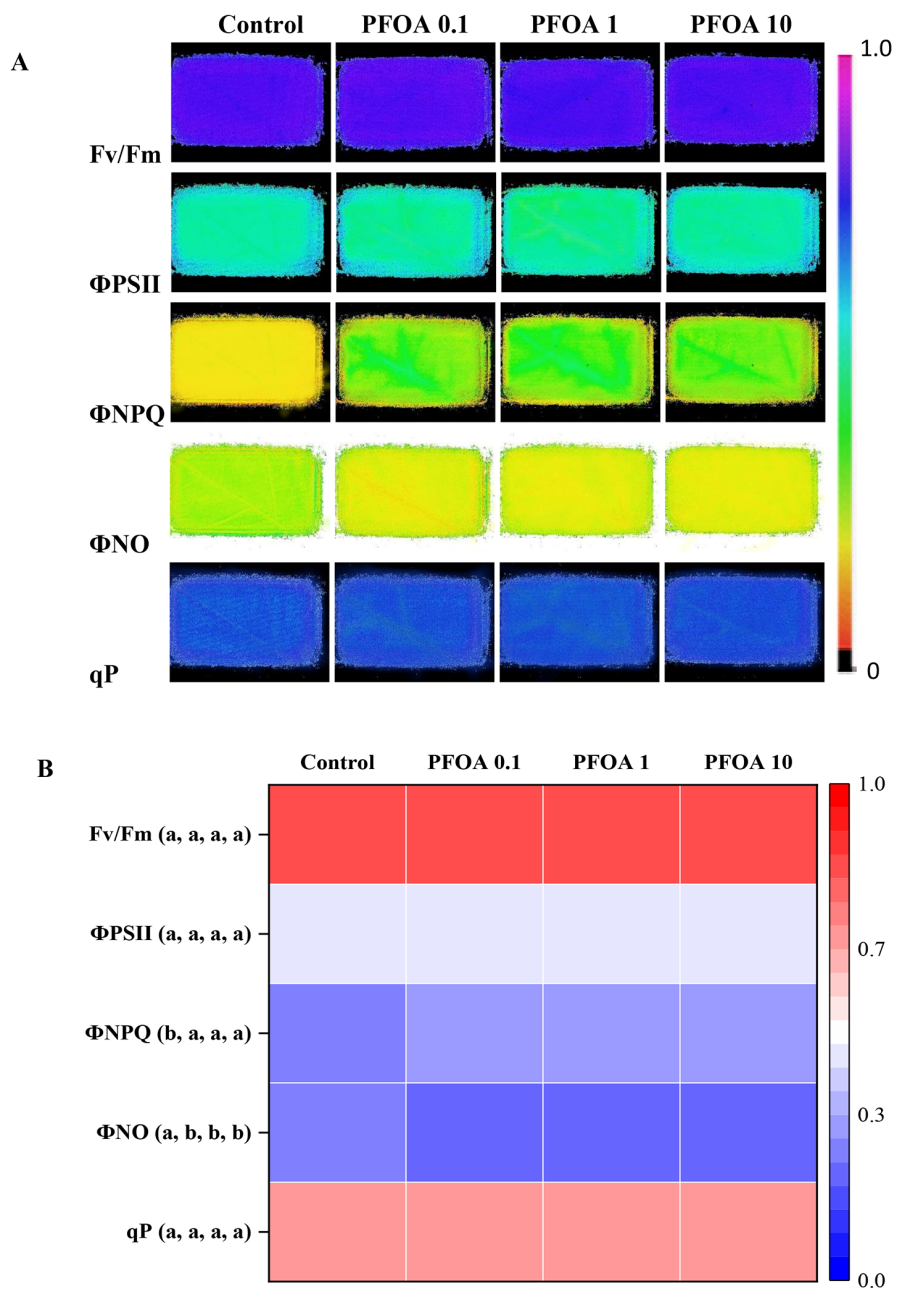
Representative images of chlorophyll fluorescence parameters (**A**) and the heatmap of their associated values (**B**) in leaves of basil (*Ocimum basilicum* L.) plants at the end of three weeks of growth in pots filled with soil with different PFOA concentrations (0 mg Kg^− 1^, Control; 0.1 mg Kg^− 1^, PFOA 0.1; 1 mg Kg^− 1^, PFOA 1; 10 mg Kg^− 1^, PFOA 10). The maximum quantum yield of PSII photochemistry (Fv/Fm), the quantum efficiency of PSII photochemistry (ΦPSII), the quantum yield of regulated (ΦNPQ) and non-regulated (ΦNO) energy dissipation in PSII and the photochemical quenching (qP) are measured with an Imaging-PAM M-series system. Data are presented as the mean of five biological replicates. A one-way analysis was applied and the different letters indicate a significant difference at *P ≤* 0.05 according to Tukey’s test

To assess the potential toxic effects of PFOA on the photosynthetic performance, measurements of leaf chlorophyll content and photochemical reflectance index (PRI) (Table 3), and analysis of chlorophyll fluorescence images (Fig. 1), were performed. These parameters have previously been identified as suitable proxies for evaluating the effects of organic pollutants on plants [60]. The data reported in this study showed that, at the end of the treatment, the chlorophyll content of basil plants exposed to different concentrations of PFOA was not significantly different from that of control plants (Table 3). Leaf chlorophyll content is one of the most important factors in determining photosynthetic potential and primary production [61] and one of the most used endpoints for assessing toxicity in plants [62]. Although many studies have shown negative effects of PFOA on chlorophyll levels when the concentration reaches a threshold [63], there are contrasting results reported in the literature on this issue. Indeed, Du et al. [37] showed a significant reduction in chlorophyll content in the leaves of cucumber plants grown in the greenhouse in soil spiked with PFOA concentrations similar to those used in the present study (i.e. 0.2 and 5 mg Kg^− 1^). In accordance with the findings of the present study, Li et al. [22] observed no alteration in chlorophyll and carotenoid levels in lettuce plants grown under hydroponics and exposed to PFOA concentrations (0.005 and 0.05 mg Kg^− 1^) lower than those used in this trial. Similarly, Pietrini et al. [39] found no impairment at the photosynthetic level in *Lemna minor* plants treated with low (0.002, 0.02 and 0.2 mg L^− 1^) PFOA concentrations under hydroponic conditions. It is notable that basil plants may be considered more tolerant to PFOA than other species, as evidenced by their ability to maintain high levels of photosynthetic pigments. To verify this finding, the photochemical reflectance index (PRI) was monitored. The PRI is related to changes in xanthophyll pigment composition. The xanthophyll cycle is a photosynthetic mechanism that dissipates excess energy as heat to protect photosystem II (PSII) under stress conditions [64]. Given the correlation between xanthophyll metabolism and photosynthesis, PRI has been proposed as an indicator of photosynthesis (observed as radiation use efficiency, RUE) [65, 66]. The data presented in this work showed that the PRI values of basil plants exposed to different concentrations of PFOA did not show any significant difference from the control plants (Table 3), thereby confirming that this contaminant did not affect the photosynthetic performance in plants of *Ocimum basilicum* L. Finally, to assess the ability of basil plants to withstand PFOA and to study the effects of the contaminant on the photosynthetic apparatus and on the spatial heterogeneity of photosynthesis, measurements of chlorophyll fluorescence imaging were performed [67–69]. Representative images of chlorophyll fluorescence parameters (F_v_/F_m_, ΦPSII, ΦNPQ, ΦNO and qP) and the heat map of their associated values in leaves of *Ocimum basilicum* L. plants measured at the end of the PFOA treatment are shown in Fig. 1. Recently, chlorophyll fluorescence image analysis has also been utilised to study the effects of PFOA on the photosynthetic activity in different plant species [22, 27, 39, 70, 71]. Overall, chlorophyll fluorescence images (Fig. 1A) showed in both dark-adapted (F_v_/F_m_) and light-adapted leaves (ΦPSII, ΦNPQ, ΦNO and qP) a nearly uniform pattern of distribution in control and PFOA-exposed plants. These findings indicate that, under our experimental conditions, treatment with PFOA did not alter the photosynthetic performance across the leaf surface. The analysis of the photochemical efficiency of photosystem II (PSII), assessed using F_v_/F_m_ in dark-adapted leaves, revealed no significant differences between the plants treated with PFOA and the control group (Fig. 1B). The F_v_/F_m_ ratio is a well-established diagnostic tool for photoinhibition [72]. In the current study, PFOA treatment had no significant impact on the abovementioned parameter, indicating no noticeable damage to the PSII reaction centre was detectable. However, ΦPSII, measuring the efficiency of PSII [73], is considered the most useful parameter for assessing photochemistry. It provides information on the electron transport rate and, unlike the F_v_/F_m_ ratio (measured under dark-adapted conditions), on the nature of photoinhibition [74]. Additionally, the coefficient of photochemical quenching qP provides valuable insights into the photochemistry, as it reveals the proportion of open PSII reaction centres [67]. Thus, to clarify the potential impacts of PFOA treatment on photochemical processes, the evaluation of the balance between light energy capture and photochemical energy utilisation in plants was conducted. This involved analysing the efficient quantum yield of PSII photochemistry (ΦPSII), the quantum yield of regulated non-photochemical energy loss (ΦNPQ), and the quantum yield of non-regulated non-photochemical energy loss (ΦNO) (Fig. 1B). The investigation into the responses of ΦPSII and qP to PFOA exposure, which were measured in light-adapted leaves, exhibited a trend that was comparable to F_v_/F_m_. No significant differences were observed between plants treated with PFOA and the control group (Fig. 1B). Regarding the quantum yield of non-photochemical energy loss, the regulated process (ΦNPQ) exhibited an increase in plants treated with PFOA in comparison to the control, whereas the non-regulated process (ΦNO) exhibited a decline in PFOA-treated plants, regardless of the PFOA concentration (Fig. 1B). Overall, the findings emphasise an initial rapid photoprotective mechanism in response to PFOA exposure. In fact, the increase in thermal energy dissipation (ΦNPQ) can occur in plants without considerable reduction in the ΦPSII values, as observed in plants exposed to different stressors [75]. Similarly, the increase in ΦNPQ can be sometimes compensated by the decrease in ΦNO, being the stress situation not strong enough and effective compensation occurs in the electron transport chain [76]. However, the maintenance of a high fraction of open PSII reaction centres (qP), indicated by the absence of statistical differences between basil plants treated with PFOA and control plants (Fig. 1B), confirms the capacity of basil plants to sustain high photosynthetic performance [77] and low “excitation pressure” on PSII [78] despite the high PFOA concentrations. The values of the chlorophyll fluorescence parameters are shown in Table S2. In accordance with the previously reported findings, Pietrini et al. [39] observed no inhibitory effects on chlorophyll fluorescence parameters (F_v_/F_m_, ΦPSII, NPQ) in *Lemna minor* L. plants exposed to varying concentrations of PFOA in hydroponics (ranging from 0.02 to 20 mg L^− 1^). In contrast, González-Naranjo et al. [70] reported a dose-dependent reduction of ΦPSII and qP in plants of *Sorghum bicolor* when exposed to different PFOA concentrations in soil (ranging from 15 to 150 mg Kg^− 1^), while Zhang et al. [27] showed a significant decrease in F_v_/F_m_, ΦPSII, qN and qP in *Arabidopsis* plants exposed to a 50 µM PFOA under hydroponic conditions.

**Table 3:** Total chlorophyll (Tot Chl) content (µg cm^− 2^) and Photochemical Reflectance Index (PRI) in leaves of basil (*Ocimum basilicum* L.) plants at the end of three weeks of growth in pots filled with soil with different PFOA concentrations (0 mg Kg^− 1^, Control; 0.1 mg Kg^− 1^, PFOA 0.1; 1 mg Kg^− 1^, PFOA 1; 10 mg Kg^− 1^, PFOA 10). In each column, similar letters represent statistically not different values (mean values ± S.E., *n* = 5; Tukey test, *P ≤* 0.05)

Treatments	Tot Chl	PRI
Control	42.04 (± 0.17) ^a^	0.088 (± 0.004) ^a^
PFOA 0.1	41.83 (± 0.57) ^a^	0.088 (± 0.002) ^a^
PFOA 1	41.26 (± 0.83) ^a^	0.092 (± 0.002) ^a^
PFOA 10	41.82 (± 1.10) ^a^	0.093 (± 0.003) ^a^

At the biochemical level, the onset of an oxidative stress process through the induction of ROS in the leaves of lettuce plants exposed to PFOA was reported by Li et al. [22], who observed an increase in malondialdehyde (MDA) content, which is one of the products of the oxidative damage to lipids. In contrast to this finding, in the present study there was no increase in the degree of lipid peroxidation measured as TBARS in basil leaves (Table 2). In fact, TBARS content in leaf tissues showed a decreasing trend as the PFOA concentration in leaves increased. In this context, it is worthy to highlight that the content of α-tocopherol, the main antioxidant of the lipid fraction of the cell [79], has an opposite trend, showing increasing values as the highest PFOA concentrations in the basil leaves occurred (Table 2). Therefore, even not statistically proven, a possible contribution of α-tocopherol in lowering the extent of the lipid peroxidation in basil leaves exposed to PFOA can be taken into account, as previously reported for the plant defense response to environmental stresses [80, 81]. Notably, to our knowledge, this is the first work dealing with α-tocopherol content evaluation in PFOA-treated plants.

**Fig. 2:**
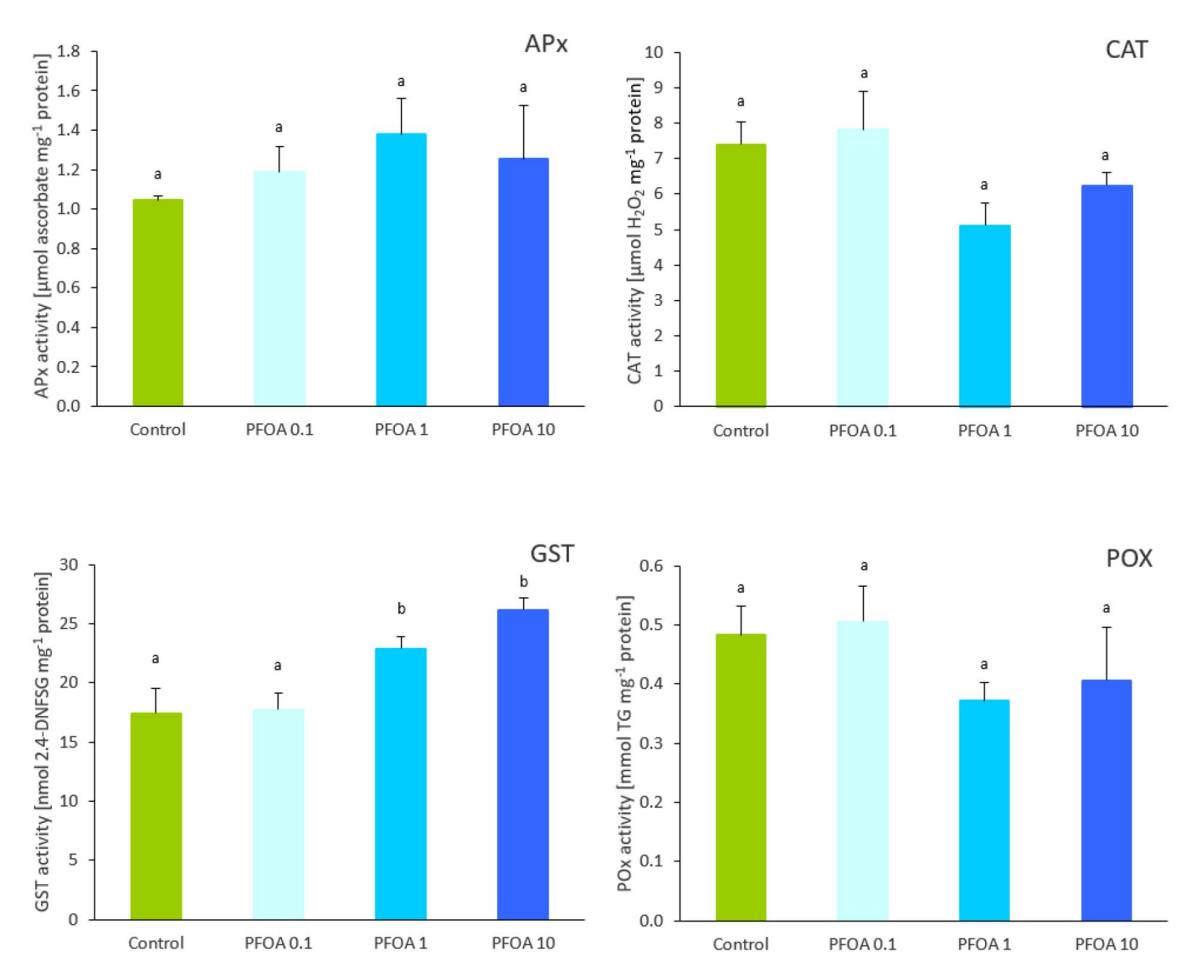
Ascorbate peroxidase (APX, µmol ascorbate mg^− 1^ protein), catalase (CAT, µmol H_2_O_2_ mg^− 1^ protein), glutathione S-transferase (GST, nmol 2.4-DNFSG mg^− 1^ protein), and guaiacol peroxidase (POX, mmol TG mg^− 1^ protein) activity in the leaves of basil plants at the end of three weeks of growth in pots filled with soil with different PFOA concentrations (0 mg Kg^− 1^, Control; 0.1 mg Kg^− 1^, PFOA 0.1; 1 mg Kg^− 1^, PFOA 1; 10 mg Kg^− 1^, PFOA 10). Different letters represent statistically different values (mean values ± S.E., *n* = 5; Holm-Sidak test, *P ≤* 0.05)

To counteract the deleterious effects possibly associated with the generation of ROS by many abiotic and biotic stressors, plants have evolved various processes involving a wide range of molecules at the enzymatic and non-enzymatic level [82]. In accordance with the lack of the increase in the oxidative stress markers (TBARS, Table 2), no activation of the antioxidant enzyme defences (APX, CAT, POX) was observed in basil leaves (Fig. 2), even in plants grown at the highest PFOA concentration in the substrate and accumulating more than 400 ng g^− 1^ dw PFOA in their tissues. Consistent with this, the non-enzymatic antioxidant response was also not stimulated in PFOA-treated plants, as previously discussed for α-tocopherol, with the concentration of TPC even decreasing with increasing PFOA concentration in the leaves (Table 2). In this regard, Li et al. [83] and Li et al. [22] reported that total leaf phenolics decreased in lettuce plants grown in a hydroponic system supplemented with 500-5,000 ng L^− 1^ PFOA or PFOS (TPC decrease: 12.1-19.3%) or with 5–50 µg L^− 1^ PFOA (TPC decrease: 27.7-33.3%), respectively. The limited literature provides contrasting data on the antioxidant response in plants exposed to PFAS. Some studies indicate that enzyme activities involved in the antioxidant reactions of plants are activated, while others suggest that these activities are inhibited. A decreasing trend in CAT activity was observed in basil leaves, especially in the PFOA 1 plants. Similarly, Omagamre et al. [84] reported a reduction in CAT activity with concomitant enrichment of genes and nonenzymatic response pathways in soybean leaves after watering the plants with water containing perfluorobutanoic acid (PFBA). Inhibition of CAT activity may lead to the accumulation of toxic H_2_O_2_ in plant tissues, which is a substrate for this enzyme. However, it is well known that H_2_O_2_ serves as a signaling molecule in the plant’s response to various types of stress, and therefore increasing its cellular concentration may initiate plant defence reactions such as enzyme activation and gene expression [85]. It cannot also be ruled out that the downward trend in CAT activity in basil leaves may be partially compensated by slightly increased APX activity, especially well presented in the same variant (PFOA 1). Such a relationship seems to be highly probable, especially due to the fact that no oxidative damage manifested by an increase in the degree of lipid peroxidation was observed. As with basil plants, Zhou et al. [25] reported a decrease in CAT activity and an increase in POX in wheat seedlings consistent with an increase in the presence of PFOA in the soil, noting that the decrease in CAT activity was correlated with the growth inhibition observed in plants treated with higher concentrations of PFOA (200–800 mg kg^− 1^).

The cultivation of basil plants in the PFOA-enriched substrate resulted in over 30 and 50% higher activity of the GST enzyme in leaves (for the PFOA 0.1 and PFOA 1 plants, respectively; Fig. 2), which was accompanied by a parallel greater accumulation of PFOA.

Glutathione S-transferases are a well-studied family of enzymes with multiple roles both in normal cellular metabolism and the detoxification of a wide variety of xenobiotic compounds, both inorganic and organic [86]. In this context, the role of GST in the detoxification pathway of organic pollutants, also known as the “green liver” concept, namely in the conjugation of the xenobiotic compound to form a polar S-glutathionylated reaction product (so-called phase II), has been extensively studied [87]. Consequently, the induction of GST activity in basil plants exposed to the highest PFOA concentrations in the substrate can be attributed to a defensive response aimed at reducing the toxic effects of PFOA accumulation in leaves, thus contributing to the prevention of damage at the growth level. To the best of our knowledge, only one paper in the literature by Zhao et al. [88] has reported evidence for the involvement of GST in plants in the degradation of PFASs, namely perfluorooctane sulfonamide (FOSA), accompanied by the absence of toxic effects at the morphological and biomass levels in soybean and pumpkin plants.

### Conclusion

The study findings indicate that, despite a notable accumulation of PFOA in basil leaves, no clear effects of this compound on plant growth and physiological performances were observed. Accordingly, no evidence of oxidative stress induction and antioxidative response was highlighted. In this regard, the increase in GST activity in PFOA-treated plants could be attributed to a detoxification process likely involved in the lack of toxicity symptoms occurring in the leaves. Given the consumption of basil leaves as a fresh herb and their utilisation in the perfume, pharmaceutical, and medical industries, this first report on the potential accumulation of PFOA in basil plants raises concerns for the safety use of this plant species when grown in or irrigated with soil or water of poor quality, respectively. This is even more relevant when there are no visual signs of toxicity in plants, as was the case in this study. Preliminary indications of this study allow considering the toxicity associated to the PFOA concentration of basil leaves grown in soil with the lowest PFOA concentration (corresponding to the soil natural background level) as negligible. Nevertheless, further studies are required to more accurately determine the impact of the presence of PFOA in the soil, and the consequent accumulation in the edible plant parts, on the food chain involving human diet.

### Electronic supplementary material

Below is the link to the electronic supplementary material.

Supplementary Material 1

## Data Availability

No datasets were generated or analysed during the current study.
