# Supplementary material for: PFOA accumulation in the leaves of basil (Ocimum basilicum L.) and its effects on plant growth, oxidative status, and photosynthetic performance
Source: BMC Plant Biol. 2024 Jun 14;24:556. doi: 10.1186/s12870-024-05269-0 (PMC11177490; doi:10.1186/s12870-024-05269-0)
Supplement: Supplementary file 1 — Supplementary Material 1 [file 12870_2024_5269_MOESM1_ESM.docx]

**Supplementary material**

**Title**:

PFOA accumulation in the leaves of basil (*Ocimum basilicum* L.) and its effects on plant growth, oxidative status, and photosynthetic performance

**Authors**:

Fabrizio Pietrini^1^, Anna Wyrwicka-Drewniak^2^, Laura Passatore^1^, Isabel Nogués^1^, Massimo Zacchini^1*^ and Enrica Donati^3^

**Institutions**:

^1^Research Institute on Terrestrial Ecosystems (IRET), National Research Council of Italy (CNR), Via Salaria km 29.300, Monterotondo Scalo, 00015 Roma, Italy

^2^University of Lodz, Faculty of Biology and Environmental Protection, Department of Plant Physiology and Biochemistry, ul. Banacha 12/16, 90-237 Lodz, Poland

^3^Institute for Biological Systems (ISB), National Research Council of Italy (CNR), Via Salaria km 29.300, Monterotondo Scalo, 00015 Roma, Italy

^*^Corresponding author:

Ph: ++390690672537

Fax: ++390690672990

E-mail: massimo.zacchini@cnr.itt

**Table S1.** Chemical properties of universal peat substrate (Select-Klasmann-Deilmann GmbH, Germany) used in the experiment.

|  |  | Chemical property | Universal peat substrate |
| --- | --- | --- | --- |
|  |  | pH | 6.00 |
|  |  | Total Organic Carbon | 40% |
|  |  | Nitrogen (N) | 210 mg/l |
|  |  | Phosphorous (P_2_O_5_) | 240 mg/l |
|  |  | Potassium (K_2_O) | 270 mg/l |
|  |  | Magnesium (Mg) | 100 mg/l |
|  |  | + trace elements  Iron added as EDTA chelate | - |

| Parameter | Leaves | Stem | Roots | F_v_/F_m_ | ΦPSII | ΦNPQ | ΦNO | qP | Tot Chl | PRI |
| --- | --- | --- | --- | --- | --- | --- | --- | --- | --- | --- |
| F-statistics | 0.946 | 2.223 | 1.354 | 2.006 | 2.910 | 6.368 | 12.246 | 3.088 | 0.198 | 0.834 |
| P-significance | 0.441 | 0.125 | 0.292 | 0.153 | 0.066 | 0.048* | <0.001** | 0.057 | 0.896 | 0.492 |

**Table S2.** F-values of one-way ANOVA which was used to test the effects of different concentrations of PFOA on growth and eco-physiological parameters of basil plants (*P*<0.05*; *P* <0.001**)

| Parameter | PFOA | TBARS | α-TOC | TPC | APX | CAT | GST | POX |
| --- | --- | --- | --- | --- | --- | --- | --- | --- |
| F-statistics | 378.28 | 0.688 | 2.437 | 4.784 | 0.622 | 2.704 | 4.269 | 0.975 |
| P-significance | <0.001** | 0.577 | 0.115 | <0.05* | 0.614 | 0.092 | <0.05* | 0.436 |

**Table S3**. F-values of one-way ANOVA which was used to test the effects of different concentrations of PFOA on PFOA, TBARS, α-Tocopherol, and Total Phenolic content, and Ascorbate peroxidase, Catalase, Glutathione S-transferase, Guaiacol peroxidase activity of basil plants (*P*<0.05*; *P* <0.001**)

**Table S4**. Biomass fresh weight (g) of different organs of basil plants at the end of three weeks of growth in pots filled with soil with different PFOA content (0 mg Kg^-1^, Control; 0.1 mg Kg^-1^, PFOA 0.1; 1 mg Kg^-1^, PFOA 1; 10 mg Kg^-1^, PFOA 10). In each column, similar letters represent statistically not different values (mean values ± S.E., n=5).

|  | Plant biomass (g) | | |
| --- | --- | --- | --- |
| Treatments | Leaves | Stem | Roots |
| Control | 20.43 (± 1.01) ^a^ | 6.36 (± 0.55) ^a^ | 6.04 (± 0.73) ^a^ |
| PFOA 0.1 | 20.75 (± 0.82) ^a^ | 7.18 (± 0.40) ^a^ | 5.58 (± 0.37) ^a^ |
| PFOA 1 | 21.00 (± 0.82) ^a^ | 7.60 (± 0.31) ^a^ | 5.84 (± 0.50) ^a^ |
| PFOA 10 | 20.72 (± 0.53) ^a^ | 7.56 (± 0.29) ^a^ | 5.66 (± 0.45) ^a^ |

**Table S5**. Chlorophyll fluorescence parameters measured in leaves of basil (*Ocimum basilicum* L.) plants at the end of three weeks of growth in pots filled with soil with different PFOA content (0 mg Kg^-1^, Control; 0.1 mg Kg^-1^, PFOA 0.1; 1 mg Kg^-^ ^1^, PFOA 1; 10 mg Kg^-1^, PFOA 10). The maximum quantum yield of PSII photochemistry (F_v_/F_m_), the quantum efficiency of PSII photochemistry (ΦPSII), the quantum yield of regulated (ΦNPQ) and non-regulated (ΦNO) energy dissipation in PSII and the photochemical quenching (qP) are measured with an Imaging-PAM M-series system. Data are presented as the mean of five biological replicates. A one-way analysis was applied, and in each column different letters indicate a significant difference at *P ≤* 0.05 according to Tukey’s test.

| Treatments | F_v_/F_m_ (rel. un.) | ΦPSII (rel. un.) | ΦNPQ  (rel. un.) | ΦNO  (rel. un.) | qP (rel. un.) |
| --- | --- | --- | --- | --- | --- |
| Control | 0.8116±0.0010 a | 0.4722±0.0032 a | 0.2592±0.0025 b | 0.2688±0.0022 a | 0.6868±0.0038 a |
| PFOA 0.1 | 0.8152±0.0006 a | 0.4522±0.0117 a | 0.3122±0.0177 a | 0.2356±0.0067 b | 0.6902±0.0079 a |
| PFOA 1 | 0.8108±0.0025 a | 0.4418±0.0052 a | 0.3218±0.0081 a | 0.2364±0.0049 b | 0.6856±0.0079 a |
| PFOA 10 | 0.8100±0.0017 a | 0.4590±0.0070 a | 0.3076±0.0103 a | 0.2334±0.0044 b | 0.7092±0.0042 a |

**Chemicals and reference standards**

Native and 1,2,3,4-^13^C_4_-labeled PFOA standard solutions (>98% purity) were purchased from Wellington Laboratories (ON, Canada). Methanol (MeOH), acetonitrile (ACN) and water (HPLC-grade) were bought from VWR International S.r.l. (Milan, Italy). Ammonium hydroxide (NH_4_OH) was acquired by Sigma-Aldrich (Steinheim, Germany). Acetic acid was supplied from J.T Baker (Milan, Italy). Formic acid was purchased from Carlo Erba (Milan, Italy). Millex-GN filters (nylon-membrane 0.20 μm, 4 mm) were purchased from Merck Millipore (Darmstadt, Germany). Oasis® HLB cartridges (6 cc, 200 mg) were obtained from Waters (Millford, MA, USA).

*Standard solution*: Stock solutions of PFOA (50 µg/mL) and 1,2,3,4-^13^C_4_-labeled PFOA (50 µg/mL) were supplied as solutions in methanol and were stored at -20°C. Intermediate (1 µg/mL) standard solutions of native and labeled PFOA were prepared by diluting the stock solutions with methanol.
